# Wilms' tumor 1 (WT1) expression and prognosis in solid cancer patients: a systematic review and meta-analysis

**DOI:** 10.1038/srep08924

**Published:** 2015-03-09

**Authors:** Xiao-wei Qi, Fan Zhang, Hong Wu, Jun-lan Liu, Bei-ge Zong, Chuan Xu, Jun Jiang

**Affiliations:** 1 Breast Disease Center, Southwest Hospital, Third Military Medical University, Chongqing, China; 2 Key Laboratory of Tumor Immunopathology, Ministry of Education of China, Chongqing, China; 3 Department of Oncology, Luzhou Medical College, Luzhou, Sichuan, China; 4 Institute of Pathology and Southwest Cancer Center, Southwest Hospital, Third Military Medical University, Chongqing, China

## Abstract

Though proposed as a promising target antigen for cancer immunotherapy, the prognostic value of Wilms' tumor 1 (WT1) in solid tumors remains inconclusive. Here, we report a systematic review and meta-analysis of the association between WT1 expression and prognosis in solid tumors. PubMed, Web of Science and Google Scholar were searched to identify studies exploring the impact of WT1 on clinical outcomes, including overall survival (OS), disease-specific survival (DSS), disease-free survival (DFS), relapse/recurrence-free survival (RFS) or progression-free survival (PFS), in solid cancer patients. Hazard ratio (HR) and 95% confidence interval (CI) were applied to assess the strength of these associations. Finally, a total of 29 eligible studies with 4090 patients were identified for qualitative analysis, and 22 studies with 3620 patients were enrolled for quantitative synthesis. Overall, positive expression of WT1 was significantly associated with worse OS (metaHR = 1.48, 95% CI = 1.11–1.97) and DFS/RFS/PFS (metaHR = 2.14, 95% CI = 1.42–3.21). Subgroup analyses showed that WT1 positive expression could independently predict unfavorable DFS/RFS/PFS (metaHR = 1.86, 95%CI = 1.04–3.35). In summary, our study suggests that WT1 may be a potential marker to predict DFS/RFS/PFS in solid tumor patients. Further studies are needed to confirm the role of WT1 expression in clinical practice.

Cancer is a major cause of mortality worldwide^1^. It is well-known that the balance between oncogenes and tumor suppressor genes is a prime determinant of normal development versus tumorigenesis^2^,^3^. Therefore, identification and characterization of these genes is of primary importance in understanding the onset and progression of cancer, and ultimately leading to recognition of potential markers and specific targets for prevention and individualized treatment of cancer.

The Wilms' tumor 1 (WT1), located at chromosome 11p13, was first cloned in 1990 as a suppressor in Wilms' tumor^4^. Subsequent research indicated that WT1 might play an oncologic role in hematologic malignancies and a variety of solid tumors, including leukemia, breast cancer, ovarian cancer, glioblastoma and soft tissue sarcoma, etc^5^,^6^,^7^,^8^,^9^. It has been shown that WT1 was overexpressed in a number of cancer cells, and more importantly, knockdown of WT1 by shRNA could induce mitochondrial damage and then inhibit malignant cell growth^10^. In addition to its prognostic role in a variety cancer types^11^,^12^,^13^,^14^,^15^, WT1 was also recognized as a promising target for immunotherapy based on its unique features^16^,^17^, and preliminary results from clinical vaccine trials revealed WT1's untapped potential to induce cancer immunity with minimal side effects^18^,^19^,^20^.

Despite the clinical development of WT1-targeted therapies, the prognostic value of WT1 expression in solid tumors remains unclear. It would clearly be desirable to explore whether the tumors manifesting WT1 expression are associated with worse outcome in patients with solid tumors. Therefore, we conducted the present systematic review and meta-analysis in order to appraise the prognostic value of WT1 in solid tumors with the goal of gaining insights into the clinical implications.

## Results

### Description of Studies

**Figure 1:**
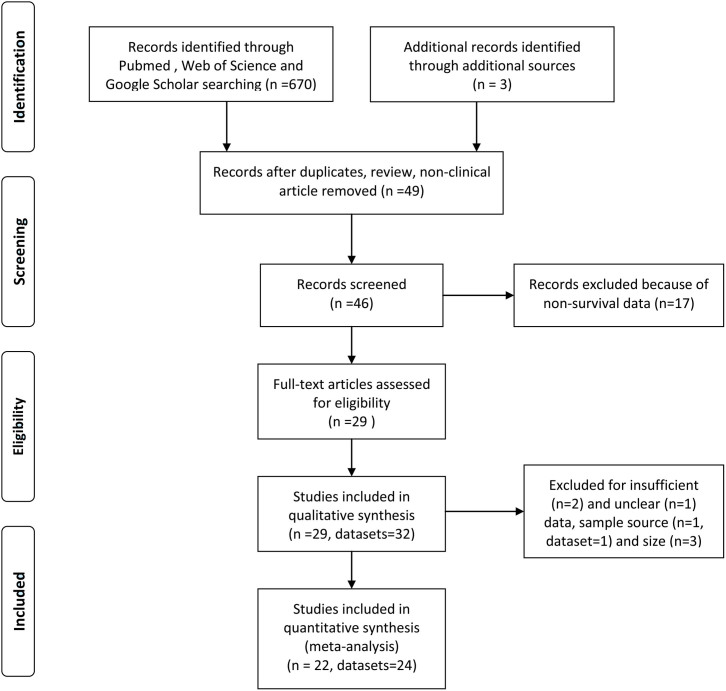
Flow diagram of study identification.

A total of 29 studies^9^,^11^,^12^,^13^,^14^,^15^,^21^,^22^,^23^,^24^,^25^,^26^,^27^,^28^,^29^,^30^,^31^,^32^,^33^,^34^,^35^,^36^,^37^,^38^,^39^,^40^,^41^,^42^,^43^ with 32 datasets and 4090 patients assessing the relationship between WT1 expression and prognosis of solid tumors were initially identified (Figure 1), and the details of selected studies are shown in Table 1. Twenty-one datasets evaluated carcinoma (9 for ovarian cancer, 5 for breast cancer, 3 for non-small-cell lung cancer [NSCLC], 2 for endometrial cancer, 1 for colorectal cancer and 1 for hepatocellular carcinoma) and 11 datasets evaluated non-carcinoma (2 for soft tissue sarcoma). Twenty-nine datasets reported an adverse impact of WT1 positive expression on prognosis regardless of the statistic power, while, 3 datasets (2 for NSCLC and 1 for soft tissue sarcoma) reported opposite results. Twenty-four datasets used antigen-based methods (21 for immunohistochemistry[IHC], 2 for ELISA and 1 for western blot), and 8 datasets used mRNA-based method (5 for reverse transcription polymerase chain reaction[RT-PCR] and 3 for gene microarray). Thirteen datasets were carried out in Europe, 14 in Asia and 5 in North of America. Twenty datasets had information on overall survival (OS), 5 had information on disease-specific survival (DSS) and 18 had information on disease-free survival (DFS)/relapse or recurrence-free survival (RFS)/progression-free survival (PFS). The median follow-up period of the 12 studies with definite follow-up time was 47 months.

For quantitative synthesis, 3 studies^38^,^40^,^41^ were excluded due to limited sample size <40, and 2 studies^42^,^43^ were rejected for insufficient data, 1 study^36^ was excluded for unclear data. Finally, 1 study^27^ and 1 dataset^32^ were rejected because the samples reported were not tissues-based. Therefore, the consolidated sample for our analysis consisted of 22 studies with 24 datasets. Among the 24 datasets, 14 addressed OS, 5 referred to DSS, and 12 pertained to DFS/RFS/PFS. A total of 3620 patients were enrolled in these studies, and the median sample size was 99, which was used as the criterion for subgroup analysis by sample size.

### Quantitative analysis of relationship between WT1 expression and OS

**Figure 2:**
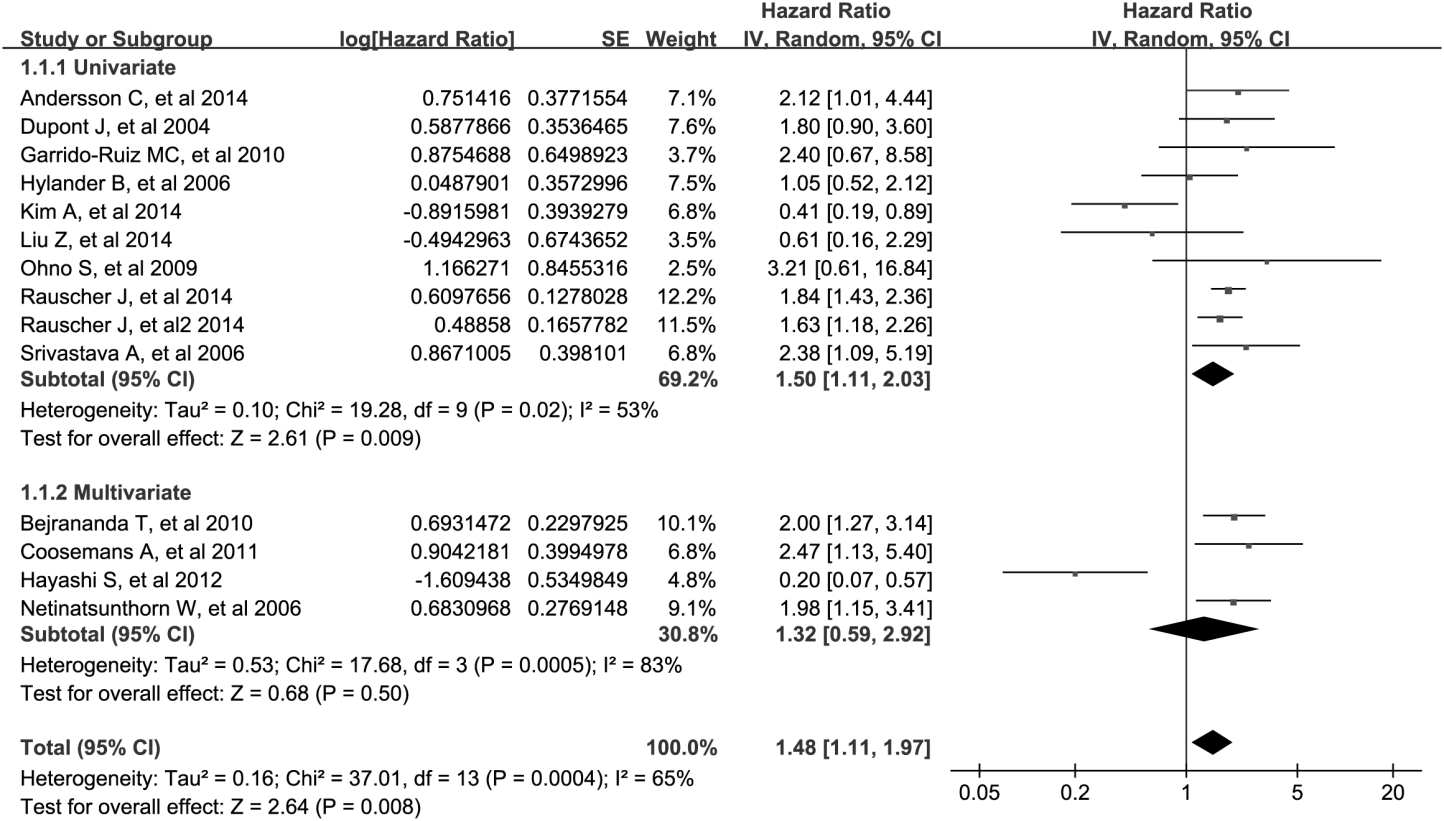
Meta-analysis of impact of WT1 expression on overall survival of patients with solid tumors. Results are presented as individual and metaHR, and 95% CI.

The combined analysis of the 14 datasets showed that WT1 positive expression was significantly associated with worse OS (meta-hazard ratio [metaHR] = 1.48, 95% confidence interval [CI] = 1.11–1.97), and it should be noted that this association only held in univariate model (metaHR = 1.50, 95%CI = 1.11–2.03), but not in multivariate analysis (metaHR = 1.32, 95%CI = 0.59–2.92) (Table 2; Figure 2). However, restricting studies to antigen-based methods, the association between WT1 positive expression and poor OS was indicated in overall (metaHR = 1.67, 95%CI = 1.27–2.21), univariate (metaHR = 1.50, 95%CI = 1.01–2.23) and multivariate (metaHR = 2.06, 95%CI = 1.50–2.83) analyses (Table 3).

Subgroup analyses by cancer type showed that WT1 positive expression had an unfavorable impact on OS for patients with ovarian cancer (metaHR = 1.57, 95%CI = 1.10–2.24), endometrial cancer (metaHR = 1.96, 95%CI = 1.04–3.72) and non-carcinoma malignancies (metaHR = 1.59, 95%CI = 1.07–2.37) (Table 2). For ovarian cancer, the prognostic value of WT1 was evaluated only in univariate model (metaHR = 1.31, 95%CI = 0.81–2.10), because only 1 study reported multivariate HR (Table 2). Subgroup analyses also suggested that the correlation of WT1 positive expression with worse OS was statistically significant among studies with using antigen-based methods (metaHR = 1.67, 95%CI = 1.27–2.21), with sample size ≥99 (metaHR = 1.52, 95%CI = 1.12–2.08), and in Europe (metaHR = 1.83, 95%CI = 1.52–2.19) as well as North of America (metaHR = 1.61, 95%CI = 1.06–2.44) (Table 2).

### Quantitative analysis of relationship between WT1 expression and DSS

**Figure 3:**
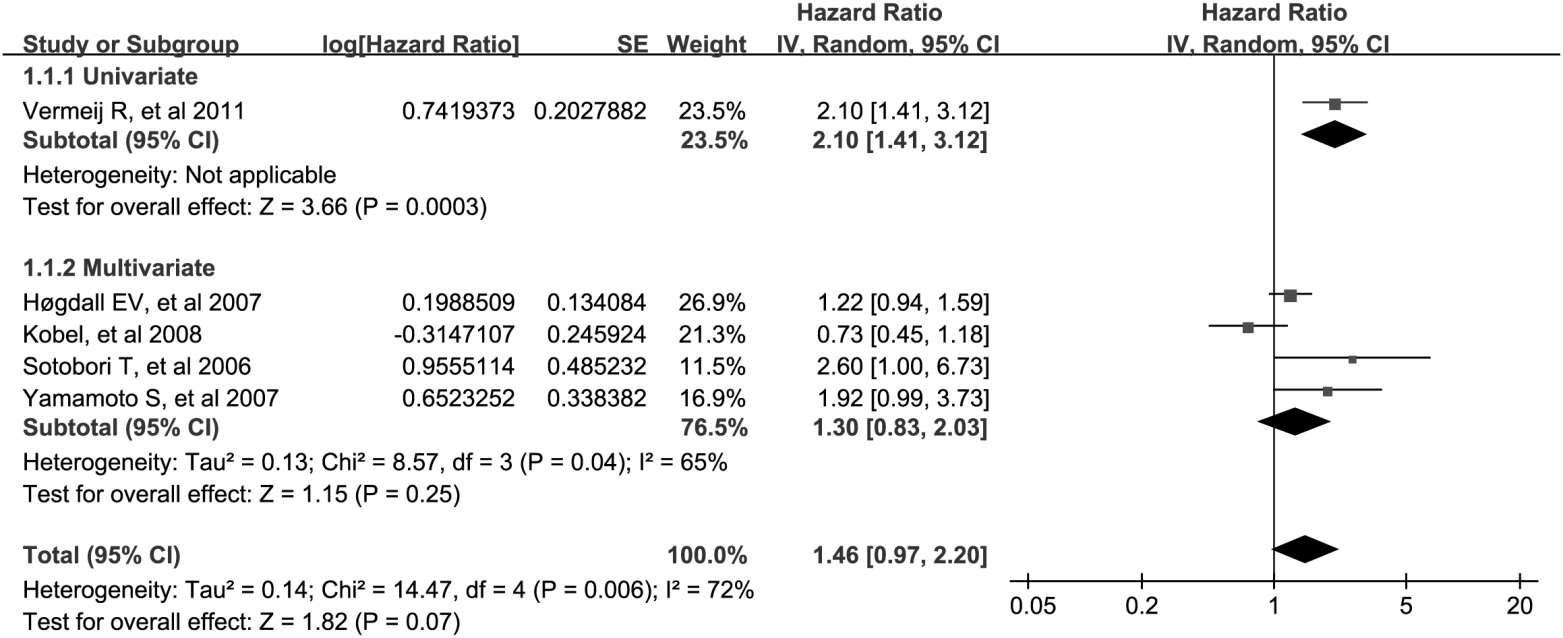
Meta-analysis of impact of WT1 expression on disease-specific survival of patients with solid tumors. Results are presented as individual and metaHR, and 95% CI.

The combined analysis of the 5 datasets showed that WT1 positive expression had borderline association with worse DSS (metaHR = 1.46, 95%CI = 0.97–2.20), and this association was also not significant in multivariate model (metaHR = 1.30, 95%CI = 0.83–2.03) (Table 2; Figure 3). In addition, restricting studies to antigen-based methods did not alter the results (for overall analysis, metaHR = 1.36, 95%CI = 0.88–2.10; for multivariate analysis, metaHR = 1.15, 95%CI = 0.93–1.43) (Table 3). Subgroup analyses also did not reveal any significant associations, except for studies carried out in Asia (metaHR = 2.12, 95%CI = 1.23–3.65) (Table 2).

### Quantitative analysis of relationship between WT1 expression and DFS/RFS/PFS

**Figure 4:**
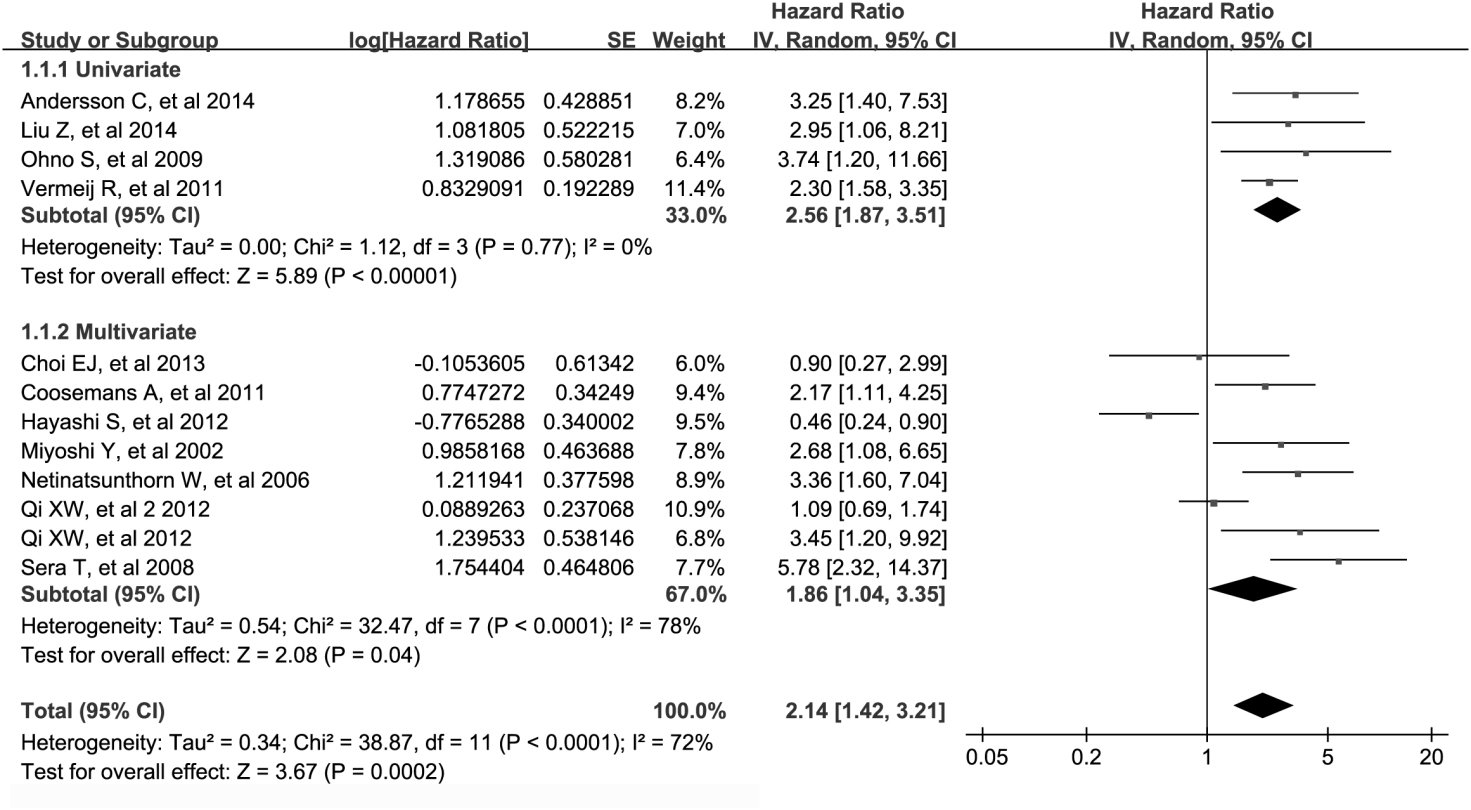
Meta-analysis of impact of WT1 expression on disease-free survival/progression-free survival/relapse or recurrence-free survival of patients with solid tumors. Results are presented as individual and metaHR, and 95% CI.

The combined analysis of the 12 datasets showed that WT1 positive expression was associated with worse DFS/RFS/PFS (metaHR = 2.14, 95%CI = 1.42–3.21), and these associations were clearly evident both in univariate (metaHR = 2.56, 95%CI = 1.87–3.51) and multivariate models (metaHR = 1.86, 95%CI = 1.04–3.35) (Table 2; Figure 4). In addition, after restricting studies to antigen-based methods, the associations were still significant in overall (metaHR = 2.61, 95%CI = 2.02–3.37), univariate (metaHR = 2.53, 95%CI = 1.82–3.51) and multivariate (metaHR = 2.73, 95%CI = 1.81–4.12) analyses (Table 3).

Subgroup analyses by cancer type showed that WT1 positive expression had an unfavorable impact on DFS/RFS/PFS for patients with carcinoma (metaHR = 2.14, 95%CI = 1.37–3.36), including ovarian cancer (metaHR = 2.61, 95% CI = 1.94–3.52). For ovarian cancer, the prognostic value of WT1 was demonstrated only in univariate model (metaHR = 2.49, 95%CI = 1.79–3.44) (Table 2). Subgroup analyses also found that the correlation of WT1 positive expression with worse DFS/RFS/PFS was significantly evident among studies using antigen-based methods (metaHR = 2.61, 95%CI = 2.02–3.37), with sample size<99 (metaHR = 3.16, 95%CI = 2.14–4.67), and in Europe (metaHR = 1.95, 95%CI = 1.52–2.49) as well as Asia (metaHR = 2.16, 95%CI = 1.01–4.62) (Table 2).

### Publication bias

**Figure 5:**
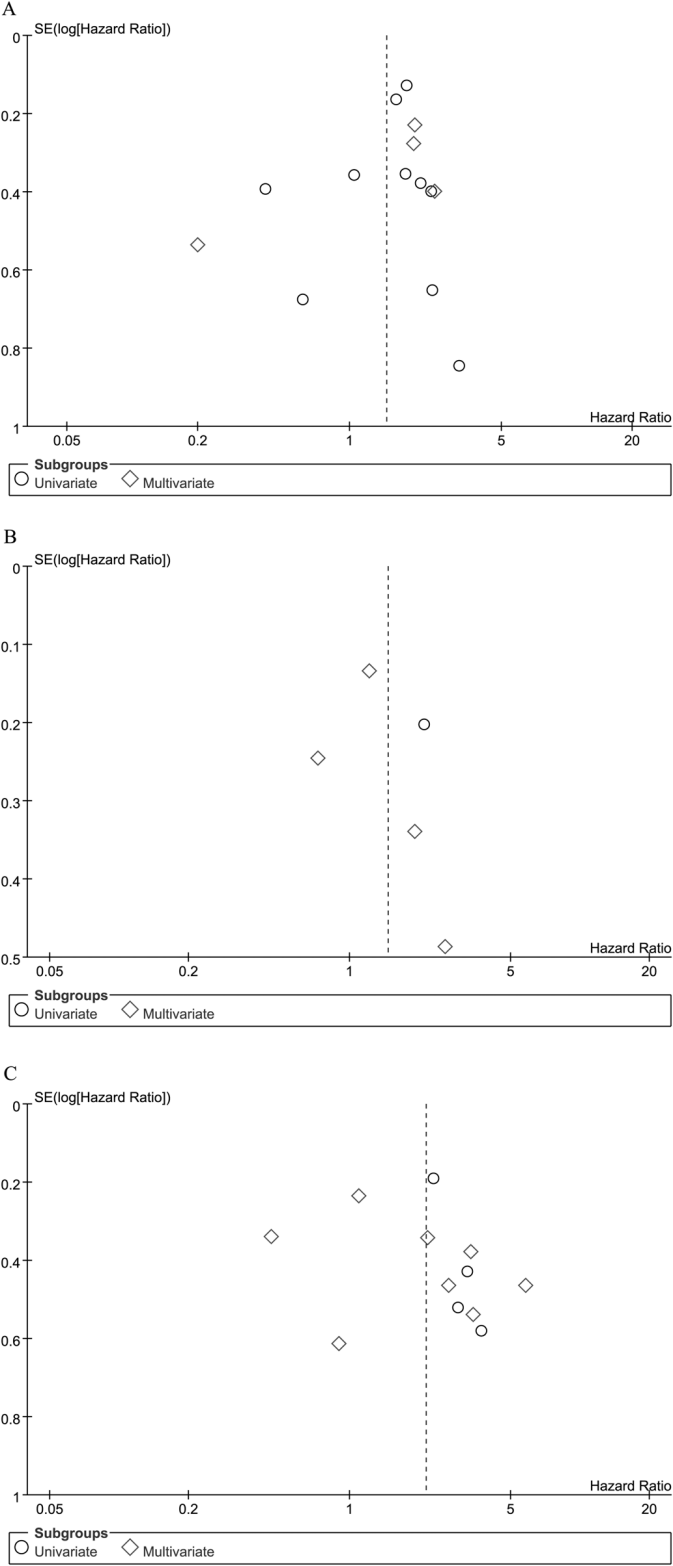
Funnel plot for the evaluation of potential publication bias in the impact of WT1 on overall survival (A), disease-specific survival (B) and disease-free survival/progression-free survival/relapse or recurrence-free survival (C) of patients with solid tumors. The funnel graph plots the log of HR against the standard error of the log of the HR (an indicator of sample size). The circles indicate the individual studies in the meta-analysis. The line in the center represents the metaHR.

The shapes of the funnel plots did not reveal evidence of obvious asymmetry for OS (Figure 5A), DSS (Figure 5B) and DFS/RFS/PFS (Figure 5C) analyses, which was confirmed by the Begg's and Egger's tests (Table 2). The results of publication bias for studies using antigen-based methods to detect WT1 expression are shown in Table 3.

### Sensitivity analysis

Sensitivity analysis was performed by sequential removal of individual studies to assess the influence of each study on the metaHRs. Results showed that metaHRs were not statistically altered (data not shown).

## Discussions

The WT1 gene, which encodes a protein consisting of four zinc finger domains at the C terminus and an glutamine and proline-rich domain at the N terminus, plays an important role in cell proliferation, differentiation, apoptosis, organ development and the maintenance of several adult tissues^44^,^45^. Though recognized as a classic tumor suppressor gene in Wilms' tumor, there is a growing body of evidence demonstrating that wild-type WT1 is expressed in a variety of tumors arising from different tissues that normally do not express WT1^46^,^47^,^48^. It is possible that WT1 could inhibit cell apoptosis by transcriptional activation and/or upregulation of proto-oncogenes^10^. Recent work has also revealed that WT1 is a key regulator in overcoming senescence downstream of KRAS signaling^49^, and is involved in the apoptotic response to cytotoxic stress^50^, further demonstrating its oncogenic effects. Moreover, WT1 could also promote invasion, migration and metastasis^43^,^51^,^52^, facilitate angiogenesis^53^,^54^ and confer drug resistance to cancer cells^55^,^56^.

Because of its implications in the etiology of cancer, clinical values of WT1 expression are the subject of increasing scrutiny. To date, the role of WT1 expression as a potential prognostic marker has been extensively explored in a number of solid tumors as listed in Table 1. In addition, considering its unique features^16^,^18^, much attention has been given to the use of WT1 peptides in eliciting an immune response. In phase I and II clinical trials for solid tumors, two patients obtained a complete remission, three had a partial remission, and five showed a decrease in tumor marker or tumor size^18^. Nevertheless, the clinical implications of WT1 expression are still largely unknown, and the prognostic value of WT1 expression in solid tumors remains controversial. Therefore, we conducted the present systemic review and meta-analysis to clarify this issue and to provide more evidence for facilitating the development of anti-WT1 target research.

The present study included a total of 29 eligible studies with 32 datasets and 4090 patients for qualitative analysis. Ovarian cancer ranked first among the reported cancer types, and IHC was most commonly used for detection. For most of these studies, positive expression of WT1 was linked with an unfavorable impact on the prognosis. However, two of three studies for NSCLC (one using ELISA, and the other one using RT-PCR) revealed that patients with high WT1 expression had a better prognosis than controls, which might be attributed to differences in detection methods as well as cellular context^49^. For meta-analysis, we excluded studies using ELISA as a detection method because the source WT1 IgG antibody in plasma was largely unclear and controversial^27^,^32^. Finally, 22 studies with 24 datasets and 3620 patients were subjected to quantitative analysis. Overall, WT1 positive expression showed significant association with worse OS and DFS/RFS/PFS, and borderline association with poor DSS, in solid cancer patients. Multivariate analyses indicated that WT1 positive expression was an independent unfavorable predictor for DFS/RFS/PFS in solid cancer patients, and the correlation became more prominent if we only focused on WT1 protein expression. Taking together, we can infer that WT1 may be of limited prognostic value for all-caused and disease-specific mortality in solid tumor patients. However, it could be a potentially useful marker to predict the risk of relapse and progression for these patients.

Since ovarian cancer was the most common cancer type reported in the current meta-analysis, we conducted overall and subgroup (uni/multivariate) analyses to explore the correlations of WT1 expression with prognosis in these patients. Results showed that WT1 positive expression could only predict poor outcomes for ovarian cancer in univariate model, but not in multivariate model. Restricting studies to antigen-based methods did not alter the results, which indicated that WT1 was not an independent prognostic marker for ovarian cancer. In fact, two large scale studies with a sample size ≥500^11^,^24^ also found that WT1 might be of limited prognostic value in ovarian cancer patients under multivariable model. There are several distinct subtypes of ovarian cancer, which develop differently and respond differently to chemotherapy^11^. At the same time, WT1 was expressed differently among these subtypes, mostly in serous carcinoma^11^,^22^,^24^,^29^,^33^. Therefore, the negative prognostic significance of WT1 in the entire cohort might reflect subtype differences in expression. It is obviously desirable to investigate the prognostic value of WT1 in each subtype individually rather than treating ovarian cancer as a homogeneous malignancy; however, since it is not feasible at present to secure the specific data regarding WT1 expression status and patients' survival data in each ovarian cancer subtype, we were unable to do this potentially important individualized analysis. Therefore, studies attempting to clarify the specific association between WT1 expression and prognosis in subtypes of ovarian cancer are strongly encouraged, as they offer the distinct possibility of advancing our insights into the clinical implications of WT1 expression in ovarian cancer.

It should be noted that there are some limitations to the analyses presented here. First, publication bias is a concern, because more positive results tended to be published, thus potentially exaggerating the association between WT1 expression and poor outcomes. Second, despite intensive effort, it was not possible to communicate with some investigators as we attempted to indentify all relevant date. In addition, because of limited number of studies, we were unable to conduct analyses for certain types of cancer, such as non-carcinoma cancer, and subtypes of specific cancer, such as ovarian cancer; in addition, we were also not allowed to do subgroup analyses specific to DFS/RFS/PFS. Third, some HRs were estimated indirectly as previously reported^57^, however, these data were less reliable than direct data from the original literature. Finally, though subgroup analysis by detection method was done, we were unable to carry out stratified analysis according to cutoff values of WT1 expression owing to great methodological variation among studies we selected here. Clearly, it is urgent to establish a uniform and validated method for assessment of WT1 expression to reduce the substantial heterogeneity currently present in clinical reporting protocols.

In summary, our study suggests that WT1 may be a potential marker to predict the risk of relapse and progression in solid cancer patients, but the indicative value of WT1 for all-caused and disease-specific mortality in these patients might be limited. Further data from large multicenter prospective studies are needed to validate the clinical importance of WT1.

## Methods

This study was performed according to the guidelines and recommendations for improving the quality of reporting of medical research such as REMARK and PRISMA^58^,^59^.

### Search strategy

A systematic literature search was performed to identify articles regarding WT1 and prognosis of solid tumors. The Pubmed, Web of Science, Google Scholar were used simultaneously, with the combination of terms “WT1 or Wilms' tumor 1 or Wilms' tumor gene 1 or Wilms' tumor protein 1 or Wilms' tumor suppressor gene 1” and “survival or prognosis or mortality or death” and ‘‘neoplasm or cancer or tumor or carcinoma’’ (up to 8 August 2014). All articles were initially reviewed by abstract and title browsing to select the relevant reports, which were subjected to further screening.

### Study selection criteria

Studies that were included in the systemic review had to meet all of the following criteria: 1) the publication explored the relation between WT1 expression and solid tumor prognosis, such as OS, DSS, DFS, RFS and PFS, 2) if one study reported multi-datasets based on different detection methods, cutoff values or populations, datasets would been recognized individually, and 3) publication language was confined to English. For qualitative meta-analysis, additional criteria were needed: 1) there were sufficient, clear, and available data to extract or estimate^57^ the individual HR and 95% CI, 2) sample size was 40 or more, 3) WT1 expression was detected in cancer tissues.

### Data extraction

Three authors (X.W. Qi, F. Zhang and H. Wu) independently extracted information using predefined criteria. The following information of each study was collected: first author, reference year, patient source, cancer type, number of patients, detection method, number and percentage of WT1 positive expression and its cutoff value, median and range of follow-up time, outcome endpoint, univariate or multivariate HR and 95%CI for WT1 positive expression (exposed group) versus WT1 negative expression (unexposed group). If univariate and multivariate HR and 95%CI were both reported, we preferred the multivariate result. For the studies with inadequate or unclear information, the authors were contacted by E-mail if possible. However, information from those authors was not forthcoming.

### Statistical analysis

MetaHR and 95% CI were applied to assess the association between WT1 expression and outcomes of solid cancer patients. Outcome endpoints were divided into three groups, OS, DSS and DFS/RFS/PFS, based on the data acquired in the current study and previous report^60^. An observed metaHR >1 implied increased hazard of all-caused mortality for OS, disease-specific mortality for DSS, and relapse/progression for DFS/RFS/PFS, respectively, in patients with WT1 positive expression. Subgroup analyses were done according to cancer type (carcinoma and non-carcinoma; for carcinoma, ovarian cancer, breast cancer, endometrial cancer and others), uni/multivariate model, detection method (antigen-based and mRNA-based), sample size (<median value and ≥median value) and patient source (Europe, Asia and North of America). Because it was the most common method used to detect WT1 expression, we conducted overall and subgroup analyses restricted to studies with antigen-based methods. In addition, we also did subgroup analysis by uni/multivariate approaches to explore whether WT1 was an independent prognostic factor for patients with ovarian cancer, since it was the most extensively studied type of solid cancer in the current meta-analysis. Heterogeneity assumption was assessed by chi-based Q-test and *I*^2^ test. The heterogeneity was considered statistically significant if *P* < 0.05. If heterogeneity across studies was not identified, the fixed-effects model (Mantel-Haenszel) was used. Otherwise, the random effects model (DerSimonian and Laird) was used. Publication bias was analyzed by performing funnel plots qualitatively, and estimated by Begg's and Egger's test quantitatively. Sensitivity analysis was carried out by removing each study at a time to evaluate the stability of the results. Two-sided *P* < 0.05 was considered statistically significant. The metaHRs and funnel plots were generated by Review Manager Version 5.3 (The Nordic Cochrane Centre, Copenhagen, Denmark). The analysis of publication bias and sensitivity was performed using STATA version 11.0 (StataCorp, College Station, TX, USA).

## Author Contributions

Conceived and designed the experiments: J.J., X.W.Q. Performed the experiments: X.W.Q., F.Z. and H.W. Analyzed the data: X.W.Q., F.Z., H.W. Contributed reagents/materials/analysis tools: X.W.Q., J.L.L., B.G.Z. and C.X. Wrote the paper: X.W.Q., F.Z. and J.J. All authors reviewed and approved the final manuscript.

## Figures and Tables

**Table 1:** Characteristics of studies exploring the relation between WT1 expression and prognosis of solid tumors

Author	Year	Patient source	Tumor type	No. of Patients	Stage/Grade	Detection method	No. of WT1 positive expression (%), cutoff value	Follow-up time media(range)	Outcomes, model
Miyoshi Y, et al	2002	Japan	Breast cancer	99	Operable cancer	RT-PCR	33/99(33.3%), ≥3.3	Median 48 m(39–60 m)	DFS, M
Dupont J, et al	2004	USA	Endometrial carcinoma	130	I–IV	IHC	34/130(26%), score≥ 4	Median 32 m(1–241 m)	OS, U
Hylander B, et al	2006	USA	Ovarian cancer	100	I–IV	IHC	78/100(78%), >5%	1–126 m	OS, U
Netinatsunthorn W, et al	2006	Thailand	Ovarian cancer	99	III–IV	IHC	50/90(56%), ≥median	1–168 m	OS/RFS, M
Høgdall EV, et al	2007	Denmark	Ovarian cancer	560	I–IV	IHC	89/560(16%), >10%	1–121 m	DSS, M
Yamamoto S, et al	2007	Japan	Ovarian cancer	119	I–IV	IHC	99/119(83%), >10%	Median 46 m(2–227 m)	DSS, M
Sera T, et al	2008	Japan	Hepatocellular carcinoma	42	I–IV	Western blot	21/42(50%), ≥1.63	1–82 m	DFS, M
Kobel, et al	2008	Canada	Ovarian carcinoma	493	I–III	IHV	174/493(35%), ≥ 5%	Median 61 m	DSS, M
Ohno S, et al	2009	Japan	Endometrial cancer	70	I–IV	IHC	31/70(44%), score ≥5	Median 61 m	OS/RFS, U
Oji Y, et al^a^	2009	Japan	NSCLC	79	I–III	ELISA	20/79(25%), median of 8777 WR U	36 m	DFS, U
Bejrananda T, et al	2010	Thailand	Colorectal cancer	157	I–IV	IHC	143/157(91%), NA	Median 116 m(77–145 m)	OS, M
Camcı, et al^b^	2011	Turkey	Breast cancer	66	I–IV	IHC	32 (48.5%), ≥1+	NA	RFS, U
Vermeij R, et al	2011	Netherlands	Ovarian cancer	229	I–IV	IHC	129/229(56%), NA	1–60 m	DSS/PFS, U
Hayashi S, et al	2012	Japan	NSCLC	98	I–IV	RT-PCR	60/98(61%), ≥0.0057	60 m	OS/DFS, M
Qi XW, et al	2012	France	Breast cancer	252	I–III	Gene microarray	106/252(42%), ≥2.1	1–200 m	DFS, M
Qi XW, et al	2012	France	Breast cancer	252	I–III	Gene microarray	7/252(3%), ≥3.1	1–200 m	DFS, M
Choi EJ, et al	2013	Korea	Breast cancer	97	T_1–3_	IHC	39/97(40), >10%	Median 63 m	DFS, M
Wu, et al^b^	2013	China	NSCLC	159	I–III	RT-PCR	79/159(49.7%), ≥3.0013	36	OS/DFS, U
Andersson C, et al	2014	Sweden	Ovarian carcinoma	50	I–IV	IHC	36/50(72%),>10%	Median 91 m(1–229 m)	OS/PFS, U
Andersson C, et al^a^	2014	Sweden	Ovarian carcinoma	52	I–IV	ELISA	26/52(50%), ≥18.8 WRU	Median 91 m(1–229 m)	OS/PFS, U
Liu Z, et al	2014	Japan	Ovarian cancer	63	I–IV	RT-PCR	18/63(29%), ≥53.94,	Median 14 m(1–66 m)	OS/DFS, U
Sotobori T, et al	2006	Japan	Soft tissue sarcoma	52	NA	RT-PCR	17/52(33%), ≥0.01	Median 45 m(9–205 m)	DSS, M
Srivastava A, et al	2006	USA	Osteogenic sarcoma	49	NA	IHC	12/49(24%), score ≥1	4–400 m	OS, U
Coosemans A, et al	2011	Belgium	Uterine sarcoma	71	I–IV	IHC	49/71(69%), score≥20	≥12 m	OS/PFS, M
Guntupalli SR, et al^c^	2013	USA	Uterine carcinosarcoma	64	I–IV	IHC	49/64(77%), score >2	Median 17 m(1–139 m)	OS/PFS, M
Kim A, et al	2014	Korea	Soft tissue sarcoma	87	I–IV	IHC	47/87(54%), score >21	Median 29 m(1–187 m)	OS, U
Chiba Y, et al^d^	2010	Japan	Glioblastoma	37	NA	IHC	25/37(68%), ≥3score	1–20 m	OS/PFS, U
Garrido-Ruiz MC, et al	2010	Spain	Melanoma	163	I–IV	IHC	39.60%, >10%	Median 120 m(1–240 m)	OS, U
Scattone A, et al^d^	2012	Italy	Diffuse peritoneal mesothelioma	31	I–III	IHC	10/31(32.3%), ≥25%	5–60 m	OS, U
Cedre's S, et al^d^	2013	Spain	Malignant pleural mesothelioma	32	III–IV	IHC	25/32(78.10%), >1%	1–40 m	OS, M
Rauscher J, et al	2014	Germany	Glioma	328	Grade II/III	Gene microarray	96/328(29%), ≥2	1–267 m	OS, U
Rauscher J, et al	2014	Germany	Astrocytoma	212	Grade II/III	IHC	68/212(32%),score ≥1	1–150 m	OS, U

Abbreviations: No. = number; RT-PCR = reverse transcription polymerase chain reaction; IHC = immunohistochemistry; U = univariate; M = multivariate; m = month; OS = overall survival; DFS = disease-free survival; PFS = progression-free survival; DSS = disease-specific survival; RFS = relapse/recurrence-free survival; NSCLC = non-small-cell lung cancer; NA = not available; WRU = WT1-reacting-unit.

^a^Study was excluded from quantitative analysis because samples were not form cancer tissue.

^b^Study was excluded from quantitative analysis because of insufficient data to estimate HR.

^c^Study was excluded from quantitative analysis because of unclear data for survival analysis.

^d^Study were excluded from quantitative analysis because of sample size <40.

**Table 2:** Meta-analysis of association between WT1 expression and prognosis of solid tumors (WT1 positive versus negative expression)

	OS				DSS				DFS/RFS/PFS			
	No.	metaHR(95%CI)	*P*_h_	*I*^2^(%)	No.	metaHR(95%CI)	*P*_h_	*I*^2^(%)	No.	metaHR(95%CI)	*P*_h_	*I*^2^(%)
Total	14	**1.48(1.11–1.97)**	0.00	65	5	1.46(0.97–2.20)	0.01	72	12	**2.14(1.42–3.21)^a^**	0.00	72
**Cancer type**												
**Carcinoma**	8	1.34(0.83–2.15)	0.00	67	4	1.36(0.88–2.10)	0.01	76	11	**2.14(1.37–3.36)**	0.00	74
Ovarian	4	**1.57(1.10–2.24)**^b^	0.21	34	4	1.36(0.88–2.10)	0.01	76	4	**2.61(1.94–3.52)**^b^	0.75	0
Univariate	3	1.31(0.81–2.10)^b^	0.19	39	1	2.10(1.41–3.12)	-	-	3	**2.49(1.79–3.44)**^b^	0.72	0
Mutivariate	1	1.98(1.15–3.41)	-	-	3	1.15(0.93–1.43)^b^	0.05	66	1	3.36(1.60–7.04)	-	-
Breast	0	-	-	-	0	**-**	**-**	**-**	4	1.43(0.99–2.06)^b^	0.10	53
Endometrial	2	**1.96(1.04–3.72)**^b^	0.53	0	0	**-**	**-**	**-**	0	**-**	**-**	**-**
**Non-carcinoma**	6	**1.59(1.07–2.37)**	0.01	67	1	2.60(1.00–6.73)	**-**	**-**	1	2.17(1.11–4.25)	**-**	**-**
**Uni/Multivariate**												
Univariate	10	**1.50(1.11–2.03)**	0.02	53	1	2.10(1.41–3.12)	-	-	4	**2.56(1.87–3.51)**^b^	0.77	0
Mutivariate	4	1.32(0.59–2.92)	0.00	83	4	1.30(0.83–2.03)	0.04	65	8	**1.86(1.04–3.35)**	0.00	78
**Detection method**												
antigen-based	11	**1.67(1.27–2.21)**	0.05	46	4	1.36(0.88–2.10)	0.01	76	7	**2.61(2.02–3.37)**^b^	0.26	23
mRNA-based	3	0.65(0.14–2.91)	0.00	89	1	2.60(1.00–6.73)	-	-	5	1.56(0.75–3.24)	0.00	78
**Sample size**												
<99	6	1.45(0.72–2.91)	0.00	71	1	2.60(1.00–6.73)	-	-	5	**3.16(2.14–4.67)**^b^	0.56	0
≥99	8	**1.52(1.12–2.08)**	0.01	64	4	1.36(0.88–2.10)	0.01	76	7	1.62(0.94–2.81)	0.00	78
**Patient source**												
Europe	5	**1.83(1.52–2.19)**^b^	0.85	0	2	1.57(0.92–2.66)	0.03	80	5	**1.95(1.52–2.49)**^b^	0.05	57
Asia	6	0.93(0.42–2.05)	0.00	83	2	**2.12(1.23–3.65)**^b^	0.61	0	7	**2.16(1.01–4.62)**	0.00	80
North of America	3	**1.61(1.06–2.44)**^b^	0.29	20	1	0.73(0.45–1.18)	-	-	0	-	-	-
**Public bias**												
Begg's test	0.38				0.81				0.45			
Egger's test	0.27				0.57				0.39			

Abbreviations: No. = number; *P*_h_ = *P*_heterogeneity_, HR = hazard ratio; CI = confidence interval; OS = overall survival; DSS = disease-specific survival; DFS = disease- free survival; PFS = progression-free survival; RFS = relapse/recurrence-free survival.

Statistically significant associations(*P*<0.05) are shown in bold if number of studies was equal or more than 2.

^a^For DFS, metaHR = 1.41, 95%CI = 1.05–1.88; for RFS, metaHR = 3.47, 95%CI = 1.87–6.45; for PFS, metaHR = 2.38, 95%CI = 1.75–3.23.

^b^If number of studies was equal or more than 2, fixed-effects model was used; otherwise, random-effects model was used.

**Table 3:** Meta-analysis of association between WT1 expression and prognosis of solid tumors (WT1 positive versus negative expression) restricted to studies with antigen-based methods.

	OS				DSS				DFS/RFS/PFS			
	No.	metaHR(95%CI)	*P*_h_	*I*^2^(%)	No.	metaHR(95%CI)	*P*_h_	*I*^2^(%)	No.	metaHR(95%CI)	*P*_h_	*I*^2^(%)
**Total**	11	**1.67(1.27–2.21)**	0.05	46	4	1.36(0.88–2.10)	0.01	76	7	**2.61(2.02–3.37)^a^**	0.26	23
**Cancer type**												
**Carcinoma**	6	**1.83(1.41–2.38)^a^**	0.66	0	4	1.36(0.88–2.10)	0.01	76	6	**2.69(2.04–3.55)^a^**	0.19	33
Ovarian	3	**1.69(1.16–2.44)^a^**	0.29	19	4	1.36(0.88–2.10)	0.01	76	3	**2.58(1.89–3.52)^a^**	0.57	0
Univariate	2	1.46(0.88–2.43)^a^	0.18	45	1	2.10(1.41–3.12)	-	-	2	**2.44(1.73–3.44)^a^**	0.46	0
Mutivariate	1	1.98(1.15–3.41)	-	-	3	1.15(0.93–1.43)^a^	0.05	66	1	3.36(1.60–7.04)	-	-
Endometrial	2	**1.96(1.04–3.72)^a^**	0.53	0	0	**-**	-	-	1	3.74(1.20–11.66)	-	-
**Non-carcinoma**	5	1.52(0.83–2.79)	0.01	72	0	**-**	-	-	1	2.17(1.11–4.25)	-	-
**Uni/Multivariate**												
Univariate	8	**1.50(1.01–2.23)**	0.03	56	1	2.10(1.41–3.12)	-	-	3	**2.53(1.82–3.51)^a^**	0.59	0
Mutivariate	3	**2.06(1.50–2.83)^a^**	0.89	0	3	1.15(0.93–1.43)^a^	0.05	66	4	**2.73(1.81–4.12)^a^**	0.09	55
**Sample size**												
<99	5	1.65(0.77–3.54)	0.00	74	0	**-**	-	-	4	**3.20(2.10–4.87)^a^**	0.40	0
≥99	6	**1.72(1.39–2.12)^a^**	0.70	0	4	1.36(0.88–2.10)	0.01	76	3	**2.31(1.67–3.19)^a^**	0.19	40
**Patient source**												
Europe	4	**1.81(1.38–2.37)^a^**	0.71	0	2	1.57(0.92–2.66)	0.03	80	3	**2.38(1.75–3.23)^a^**	0.73	0
Asia	4	1.41(0.66–3.04)	0.00	79	1	1.92(0.99–3.73)	-	-	4	**3.23(2.02–5.18)^a^**	0.11	50
North of America	3	**1.61(1.06–2.44)^a^**	0.29	20	1	0.73(0.45–1.18)	-	-	0	-	-	-
**Public bias**												
Begg's test	0.44				1.00				0.76			
Egger's test	0.92				0.86				0.65			

Abbreviations: No. = number; P_h_ = P_heterogeneity_, HR = hazard ratio; CI = confidence interval; OS = overall survival; DSS = disease-specific survival; DFS = disease- free survival; PFS = progression-free survival; RFS = relapse/recurrence-free survival.

Statistically significant associations(*P*<0.05) are shown in bold if number of studies was equal or more than 2.

a If number of studies was equal or more than 2, fixed-effects model was used; otherwise, random-effects model was used.
